# Highly Bioavailable Curcumin, but Not Native Curcumin, Combined With Exercise Improves Cognitive Function in Mice

**DOI:** 10.1002/fsn3.70219

**Published:** 2025-04-30

**Authors:** Tomoya Suzuki, Chisa Fushimi, Hiroki Aoyama, Atsuhiro Kishimoto, Yasuhiro Katsuura, Takanori Tsuda

**Affiliations:** ^1^ School of Bioscience and Biotechnology and Graduate School of Bioscience and Biotechnology Chubu University Kasugai Aichi Japan; ^2^ Therabiopharma Inc. Kawasaki Kanagawa Japan

**Keywords:** brain‐derived neurotrophic factor, cognitive improvement, curcumin formulation, exercise, hippocampus

## Abstract

Curcumin (Cur) has been found to improve cognitive function at high doses. However, contradictory findings have also been reported, mainly due to its low water solubility and very low bioavailability. We previously used an amorphous individual dispersion technique to develop a novel highly bioavailable Cur formulation (HC) for use as a dietary supplement. Because exercise also improves brain function, food‐derived factors and exercise might synergize to boost cognitive function. After confirming that HC alone (4.5 mg Cur/kg [equivalent to 15 mg HC/kg]) did not improve cognitive function, we demonstrated that the combination of HC, but not native Cur, and voluntary exercise for 4 weeks significantly improved spatial memory and learning in mice in the novel location‐recognition and passive avoidance tests, even though exercise alone did not have this effect. This effect was related to elevated expression of brain‐derived neurotrophic factor via activation of two related pathways. HC, and not native Cur, may help to improve learning and memory functions in both younger and older adults as a dietary supplement during exercise.

## Graphical abstract

Highly bioavailable curcumin (HC), but not native curcumin, combined with exercise improves cognitive function in mice, even though exercise alone does not have this effect. The Sirt1/PGC‐1α/FNDC5/BDNF pathway and activation of CREB via CaMKII enhance the upregulation of BDNF with exercise and HC. Our findings suggest that HC may help to improve learning and memory functions in both elderly and younger people as a dietary supplement during exercise.

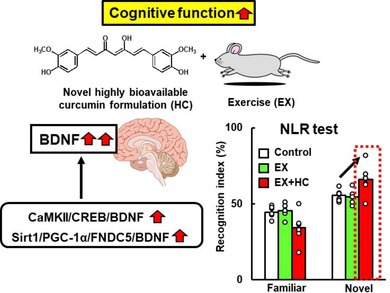

**Abbreviations**
BDNF: brain‐derived neurotrophic factor
CaMKII: calcium/calmodulin‐dependent protein kinase II
CREB: cAMP‐response element‐binding protein
Cur: curcumin
ERK: extracellular signal‐regulated kinase
ERRα: estrogen‐related receptor α
FNDC5: fibronectin type III domain‐containing 5
HC: highly bioavailable Cur formulation
NC: native Cur
NLR: novel location‐recognition
PA: passive avoidance
PGC‐1α: peroxisome proliferator‐activated receptor γ coactivator‐1α
PSD95: postsynaptic density protein 95
Sirt1: sirtuin 1

## 1 Introduction

Curcumin (Cur) is a yellow pigment present in the flowering plant turmeric ( *Curcuma longa* ) and has been linked with improved brain function. For example, Cur administration was shown to ameliorate cognitive decline in an animal model of Alzheimer's disease (Reddy et al. 2018). In humans, Cur intake has been reported to boost cognition from middle age onward (Cox et al. 2015, 2020; Kuszewski et al. 2020). However, these studies have typically involved high doses (Oz et al. 2015; Mehla et al. 2010) and some conflicting results have been reported (Santos‐Parker et al. 2018; Ringman et al. 2012). This is mainly because Cur has low water solubility with very low bioavailability (Tønnesen et al. 2002; Yang et al. 2007; Mirzaeia et al. 2017). For this reason, various highly bioavailable Cur formulations are being designed and developed for use as dietary supplements to reduce the functional dose (Tsuda 2018).

We recently developed a novel Cur formulation, using an amorphous individual dispersion technique (Sunagawa et al. 2021). In general, most Cur particles are excreted intact from the body due to their large and rigid crystalline state, but the novel Cur formulation overcomes this problem by making the crystal structure easier to collapse (amorphization) through disordered alignment, resulting in smaller particles. This formulation exhibits higher bioavailability compared with other Cur formulations or native Cur (NC) (Sunagawa et al. 2021) and has been shown to ameliorate knee osteoarthritis and improve immune function in humans (Nakagawa et al. 2023; Kishimoto et al. 2021).

Exercise confers various health benefits in humans, and there is increasing evidence that exercise has positive effects on brain function. Recent reports have shown that exercise prevents cognitive decline or improves brain function in people with mild cognitive impairment or even with Alzheimer's disease (Thomas et al. 2020; Yu et al. 2021). Accordingly, it is possible that food‐derived factors and exercise synergize to confer exercise‐associated health benefits. In earlier work, we showed that the combination of food‐derived factors, such as amino acids or soy protein, with exercise induces beige adipocytes in inguinal white adipose tissue and that this approach can help to suppress body fat accumulation (Kojima et al. 2021; Kato and Tsuda 2023). More recently, we demonstrated that the combination of a low‐dose Cur and exercise significantly induced the development of beige adipocytes, unlike exercise or Cur alone (Tanahashi et al. 2023). In addition, another research group reported that the combination of the Cur formulation and exercise improved follicular dysfunction in rats (Zhang et al. 2021) and cardiac function in postmenopausal women (Sugawara et al. 2012). However, it remains unclear whether the combination of Cur and exercise efficiently improves cognitive function and the underlying molecular mechanism. Considering the above, in this study, we examined the hypothesis that the combination of exercise and the highly bioavailable Cur formulation (HC), but not NC, would effectively improve cognitive function and that a lower dose of Cur could be used to boost cognitive function via combination with exercise.

In the present study, we show that exercise combined with HC, but not NC, significantly improves cognitive function in mice. Moreover, we found that the mechanism underlying this effect is associated with upregulation of brain‐derived neurotrophic factor (BDNF, a protein that is essential for hippocampus‐associated learning and memory) via activation of two related pathways. Our findings suggest that HC could be used as a dietary supplement during exercise to boost learning and memory functions in both younger and older adults.

## 2 Experimental Section

### 2.1 Chemicals and Antibodies

HC (Lot No. 200901) and placebo (control) (Lot No. 200626) were obtained from Therabiopharma Inc. (Kanagawa, Japan) (Sunagawa et al. 2021). HC comprised 31% Cur, 47% modified starch, 19% crystalline cellulose, 2% calcium stearate, and 1% silicon dioxide. NC (> 99% purity) was provided by Fujifilm Wako Pure Chemical Corporation (Osaka, Japan). The placebo comprised 71% crystalline cellulose, 2% calcium stearate, 1% silicon dioxide, and 25.9% yellow food coloring. The Supporting Information (Supporting Materials and Methods) details the antibodies used in this study.

### 2.2 Animal Experiments

The Animal Experiment Committee of Chubu University approved the animal experiments in this study. In addition, the relevant institutional guidelines were adhered to for the care and use of all mice (Permission No. 202110016).

### 2.3 Dosage Information/Dosage Regimen

Placebo, NC, and HC were dissolved in a saline solution and administered to mice daily by gavage for 4 weeks at the following doses: placebo (control), 10.5 mg/kg body weight; NC, 4.5 mg Cur/kg; and HC, 4.5 mg Cur/kg (equivalent to 15 mg HC/kg), with reference to our previous study (Nishikawa et al. 2018). In addition, preliminary work conducted to determine the doses of NC and HC found that they did not significantly affect food intake or body weight. For a 60‐kg human, the equivalent dose was determined to be 0.36 mg Cur/kg body weight (Nair and Jacob 2016).

### 2.4 Determination of Plasma Cur Levels After Single Oral Administration of NC or HC

After a 16‐h fast, 12‐week‐old ICR mice (all male) were randomly divided into two groups: a NC group (4.5 mg Cur/kg, *n* = 4) and a HC group (4.5 mg Cur/kg [equivalent to 15 mg HC/kg], *n* = 4). The animals received single oral doses of NC or HC by gastric intubation. Blood samples were collected from isoflurane‐anesthetized mice using a syringe containing heparin. The blood was isolated after NC or HC administration at 0.25, 0.5, 1, and 2 h after loading, centrifuged to isolate plasma, and stored in a −80°C freezer until required. Plasma Cur concentrations were measured as described previously with modifications (Sunagawa et al. 2021). Briefly, 20 μL of each plasma sample was placed in a brown microtube, to which was added 100 μL of 0.1 M acetate buffer (pH 5.0) and 10 μL of β‐glucuronidase solution at 96,000 units/mL (Fujifilm Wako Pure Chemical Corporation). After 1 h at 37°C to hydrolyze the Cur conjugates, 12 μL of the internal standard solution (fluorinated‐Cur at 500 ng/mL) and 0.5 mL of chloroform were added. In addition to mixing with a vortex mixer for 30 s, the mixture was sonicated for another 20 min. Next, after centrifugation for 5 min at 11,800 × *g*, the organic layer was separated into a microtube. The extraction process described above was repeated. Using a centrifuge concentrator, the organic layer was dried by evaporation. The dry sample was dissolved in 120 μL of 50% acetonitrile with 0.1% formic acid and centrifuged for 5 min at 11,800 × *g*. Two microliters of the supernatant was injected into a HPLC‐MS/MS system composed of an Elute UHPLC chromatography system (Bruker Japan K.K., Yokohama, Japan) and micrOTOF compact (Bruker Japan K.K.) with (+) electrospray ionization to measure the Cur concentration. Sample separation was conducted with an Atlantis T3 column (2.1 × 150 mm, 3 μm; Waters Corporation, Milford, MA) and a gradient of binding solvent (0.1% formic acid in water; solution A) and elution solution (0.1% formic acid in acetonitrile; solution B) at a 0.2‐mL/min flow rate and 40°C column temperature. The solvent gradient was varied according to the following conditions: 40% (0.00–0.50 min), 40%–75% (0.50–2.00 min), 75%–99% (2.00–2.01 min), 99% (2.01–6.00 min), 99%–40% (6.00–6.01 min), and 40% (6.01–9.00 min) in B. The mass spectrometer was operated in multiple reaction monitoring mode using electron spray thermos ionization. The monitored transitions (from precursor to product) were m/z 369.1 → 177.0 (m/z) for Cur and 387.1 → 177.0 (m/z) for fluorinated‐Cur. The total plasma Cur concentration (including both free and conjugated Cur) was measured.

### 2.5 Effects of Oral NC or HC Intake Without Exercise on Cognitive Function

ICR mice (male; 7 weeks old; Japan SLC, Hamamatsu, Japan) were housed in an experimental animal room with lights on from 08:00 to 20:00 and maintained at 23°C ± 3°C. Animals had ad libitum access to water and to standard CE‐2 laboratory chow (CLEA Inc., Tokyo, Japan) (Kojima et al. 2021; Kato and Tsuda 2023; Nishikawa et al. 2018). Following a 1‐week acclimatization period, the mice were randomly assigned to the control (placebo), NC, or HC group. During the experimental period, all mice were given ad libitum access to semi‐purified standard chow (based on a partial modification of the AIN‐93G diet) (Nishikawa et al. 2019; Reeves et al. 1993) and drinking water. In the last week before the end of the experiment, behavioral studies to assess cognitive function were conducted as shown below in 2.7 and 2.8. A brief timeline for the experiments is shown in Figure 2A.

### 2.6 Effects of HC Combined With Voluntary Exercise on Cognitive Function

ICR mice (male; 7 weeks old; Japan SLC) were maintained as reported above. Following a 1‐week acclimatization period, the animals were assigned to the control group, an exercise group, or an exercise + HC group. The control and exercise groups were administered placebo, whereas the exercise + HC group was administered HC. Mice in the exercise and exercise + HC groups were individually housed with uninterrupted access to a computer‐monitored angled voluntary running wheel (outer diameter of the rotating wheel, 15.5 cm; LCW‐M4, Melquest Ltd., Toyama, Japan) throughout the experimental period. Numbers of rotations were recorded through a counting system (CNT‐10, Melquest Ltd.) that was located on the outside of the cage to calculate the running distance per mouse. In the last week before the end of the experiment, behavioral studies to assess cognitive function were performed as described in 2.6 and 2.7. A brief timeline for the experiments is shown in Figure 3A. When the experiments were completed, the mice were anesthetized with isoflurane and blood samples were collected with a heparin‐containing syringe. The plasma was isolated by centrifugation. The hippocampus was also isolated and then immediately frozen using liquid nitrogen for storage in a −80°C freezer until required. To detect proteins, tissue aliquots were homogenized and subjected to immunoblotting, with glyceraldehyde‐3‐phosphate dehydrogenase as the loading control, as detailed in our prior studies (Kojima et al. 2021; Kato and Tsuda 2023; Esaki et al. 2023). The western blot analysis protocol is summarized in the Supporting Information.

### 2.7 Novel Location‐Recognition Test

A novel location‐recognition (NLR) test was performed as previously described (Vogel‐Ciernia and Wood 2014; Denninger et al. 2018). The NLR test is a straightforward and effective method for assessing hippocampus‐dependent spatial memory (Vogel‐Ciernia and Wood 2014; Denninger et al. 2018). Briefly, mice were given 5‐min habituation sessions for 3 consecutive days prior to acquisition sessions in an arena (57 cm × 57 cm × 40 cm). After the habituation sessions, two identical objects were placed on the same line in the arena and the time spent by mice searching for the objects was measured for 5 min/mouse. Testing sessions were conducted 24 h after the acquisition trials. In the testing sessions, one of the objects in the arena was placed on the diagonal and the time spent by mice examining the novel location object was measured for 5 min/mouse. A video camera placed above the arena was used to record the acquisition and testing trials. Analysis was conducted using a video tracking system (SMART 3.0, Panlab, S.L., Harvard Apparatus Spain, Barcelona, Spain). All objects were cleaned with 70% ethanol to eliminate odor cues between trials. The analyses of exploration time (in seconds) were based on the time spent exploring the novel and familiar locations. The recognition indices (%) of both of these locations were determined by dividing the time spent by each mouse examining the location by the overall investigation time in order to calculate how thoroughly each mouse examined the novel location.

### 2.8 Passive Avoidance Test

Passive avoidance (PA) testing was conducted using a step‐through PA apparatus cage (MPB‐M030; Melquest Ltd.) (Corpuz et al. 2019). The PA test measures learning and memory ability, using avoidance behavior of previously experienced aversive stimuli as a measure of memory. For the training session, mice were moved to a bright compartment for an exploration time of 20 s. Then, a guillotine door was raised to permit entry into a dark compartment. At this point, the door was closed and an electric foot shock was administered (0.3 mA, 2 s). The test sessions were repeated after 24 h but with no foot shock. Latencies (in seconds) to enter the dark compartment were measured for a maximum of 480 s.

### 2.9 Statistical Analysis

For comparison among three groups, data were analyzed using a Tukey–Kramer test (Figures 2, 3, 4, 5, 6; Figures S1 and S2, and Tables S1 and S2). The differences between the means of two groups were analyzed by a Student's *t*‐test (Figure 1). All results are reported as means ± SEM. *p* < 0.05 was set as significant.

## 3 Results

### 3.1 Plasma Cur Concentrations After Single Oral Administration of NC or HC

**FIGURE 1:**
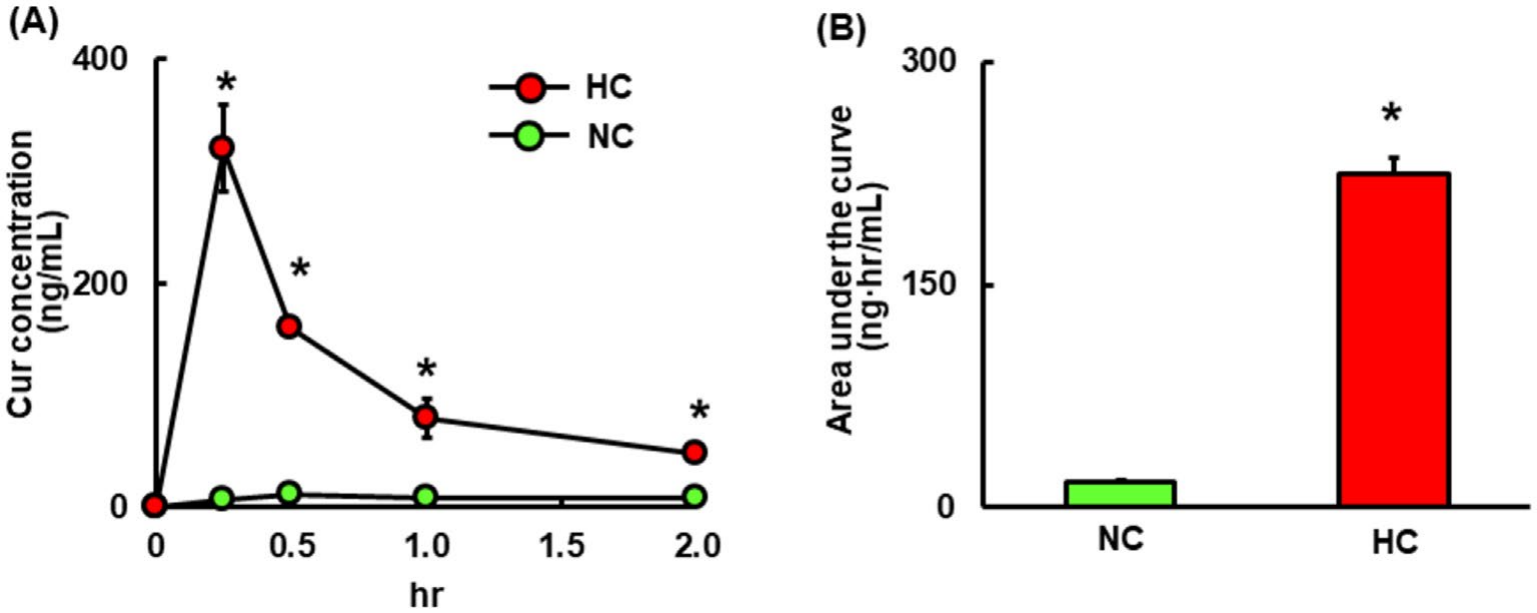
Plasma Cur concentrations in mice after single oral doses of NC (4.5 mg Cur/kg) or HC (4.5 mg Cur/kg [equivalent to 15 mg HC/kg]). Data are presented as means + SEM (*n* = 4). *Mean values are significantly different from those of the NC group (*p* < 0.05).

We first confirmed significantly higher bioavailability of HC compared with NC in ICR mice of the same age. Although plasma Cur levels were extremely low with NC, HC administration resulted in significantly elevated Cur levels (a roughly 13‐fold higher absorption vs. NC from 0 to 2 h in the area under the curve) (Figure 1).

### 3.2 Administration of NC and HC Does Not Significantly Improve Cognitive Function in Mice

**FIGURE 2:**
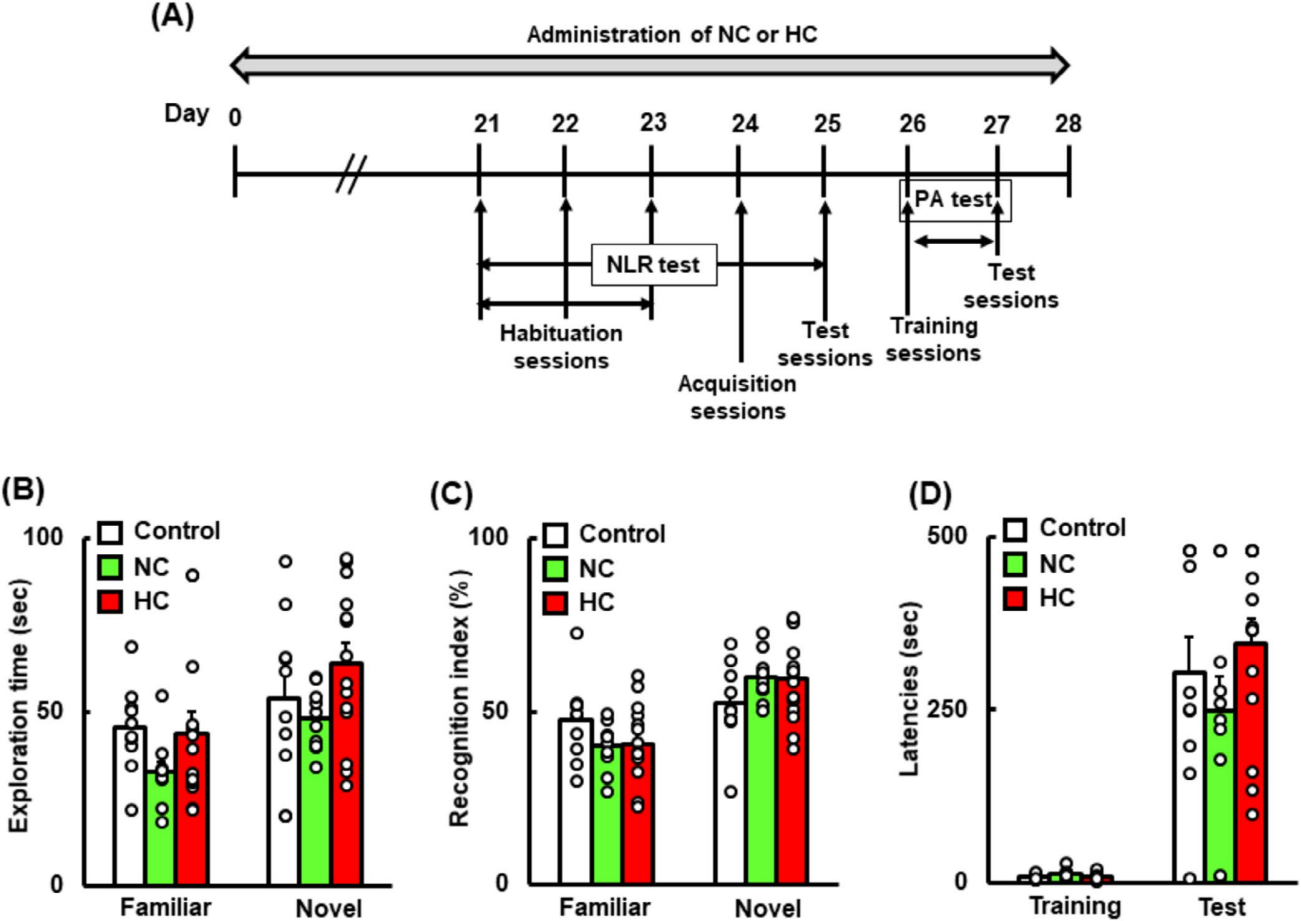
Schematic experimental design (A), time spent exploring the familiar and novel locations (in seconds) (B), and the recognition index (%) (C) after the NLR test and latencies (in seconds) after the PA test (D) in the control, NC, and HC groups. Data are presented as means + SEM (*n* = 10–14).

We next examined whether administration of NC and HC improved cognitive function without exercise in mice. Final body weight and total food intake exhibited no differences among the mouse groups (Table S1). As shown in Figure 2, neither exploration time (in seconds) nor the recognition index (%) in the NLR test significantly differed among the groups. In addition, in the PA test, the latencies to enter the dark compartment exhibited no significant increase in either the NC or HC group compared with the control.

### 3.3 HC, but Not NC, Combined With Exercise Significantly Improves Cognitive Function in Mice

**FIGURE 3:**
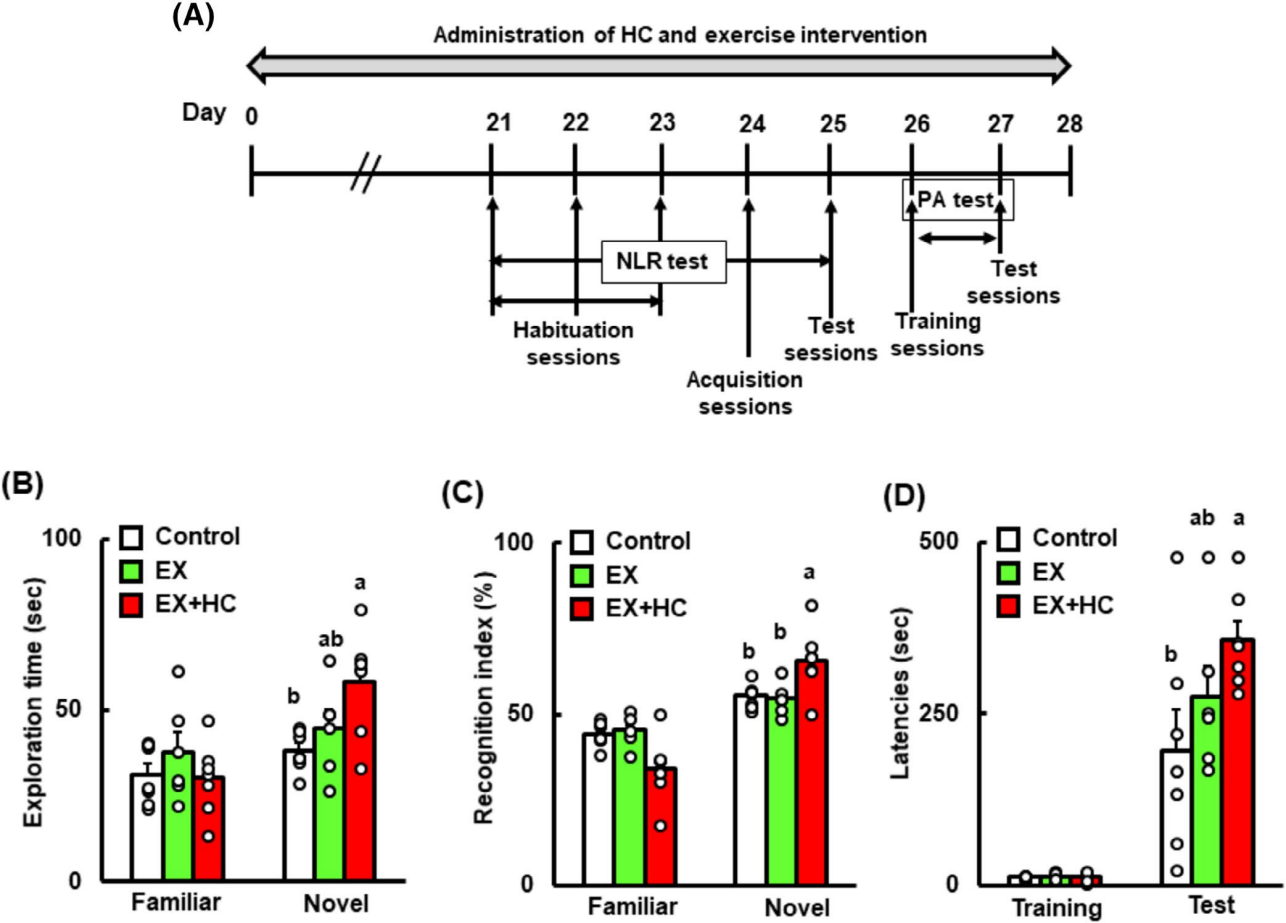
Schematic experimental design (A), time spent exploring the familiar and novel locations (in seconds) (B), and the recognition index (%) (C) after the NLR test and latencies (in seconds) after the PA test (D) in the control, EX, and EX + HC groups. Data are presented as means + SEM (*n* = 6–7). Values without a common letter are significantly different at *p* < 0.05. EX, exercise.

Next, we investigated whether HC or NC, combined with exercise, improved cognitive function at the dose (4.5 mg Cur/kg) that did not significantly affect cognitive function. Although total food intake did not differ among the groups, final body weight was significantly reduced in both the exercise and exercise + HC groups compared with the control (Table S2). Total running distance did not differ between the exercise and exercise + HC groups (Table S2). The exploration time and recognition index results in the NLR test were significantly higher in the exercise + HC group, although exercise alone had no effect on these indices (Figure 3B,C). Furthermore, the latencies in the PA test were significantly higher in the exercise + HC group versus the control group (Figure 3D).

Because exercise + HC was observed to improve cognitive function, we investigated whether NC combined with exercise had the same effect. To do so, NC (4.5 mg Cur/kg) was administered to mice and combined with exercise for 4 weeks under the same experimental conditions as for HC. The NLR test results showed that exercise + NC and exercise alone did not significantly improve cognitive function compared with the control (Figure S1). The results of the PA test also indicated that the test latencies showed no difference among the groups (Figure S1).

### 3.4 HC Combined With Exercise Significantly Induces the Hippocampal Expression of BDNF Protein in Mice

**FIGURE 4:**
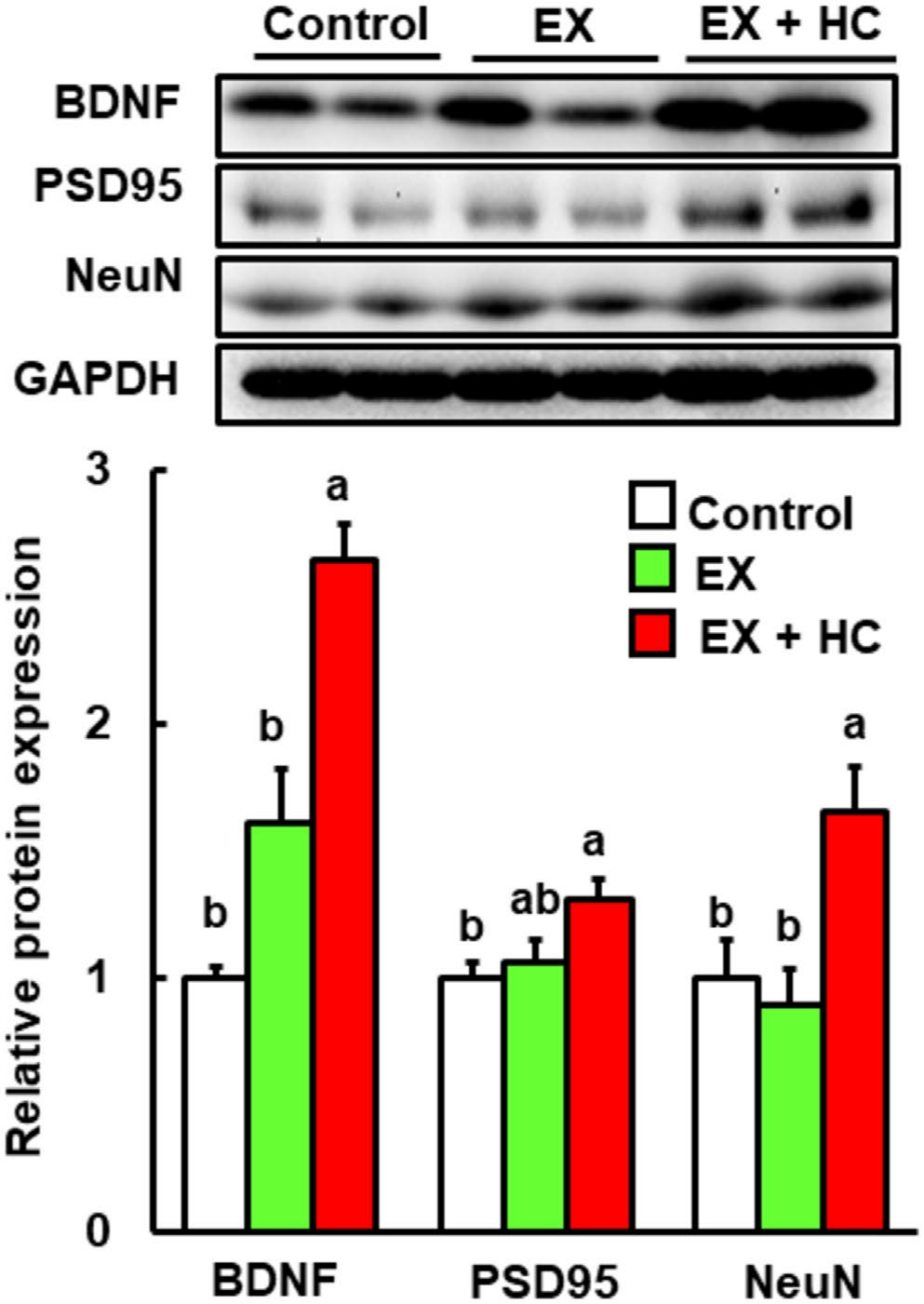
Protein expression levels of BDNF, PSD95, NeuN, and GAPDH in the hippocampus in the control, EX, and EX + HC groups. Protein levels are expressed as fold‐change relative to the control (= 1) after normalization to the GAPDH protein level. Data are presented as means + SEM (*n* = 6–7). Values without a common letter are significantly different at *p* < 0.05. EX, exercise; GAPDH, glyceraldehyde‐3‐phosphate dehydrogenase; NeuN, neuronal nuclei.

The significant improvement in cognitive function in the exercise + HC group begged the question of how the combination of HC with exercise improved cognitive function. BDNF is a crucial protein that is essential for hippocampus‐associated learning and memory (Tyler et al. 2002). The hippocampal level of BDNF protein was significantly higher in the exercise + HC group but not in the exercise alone group compared with the control (Figure 4). In addition, postsynaptic density protein 95 (PSD95), which is a plasticity‐related protein, and neuronal nuclei, a marker of neurogenesis, were also significantly higher in the exercise + HC group (Figure 4).

### 3.5 Upregulation of BDNF With Exercise + HC Is Associated With Two Related Pathways in the Mouse Hippocampus

**FIGURE 5:**
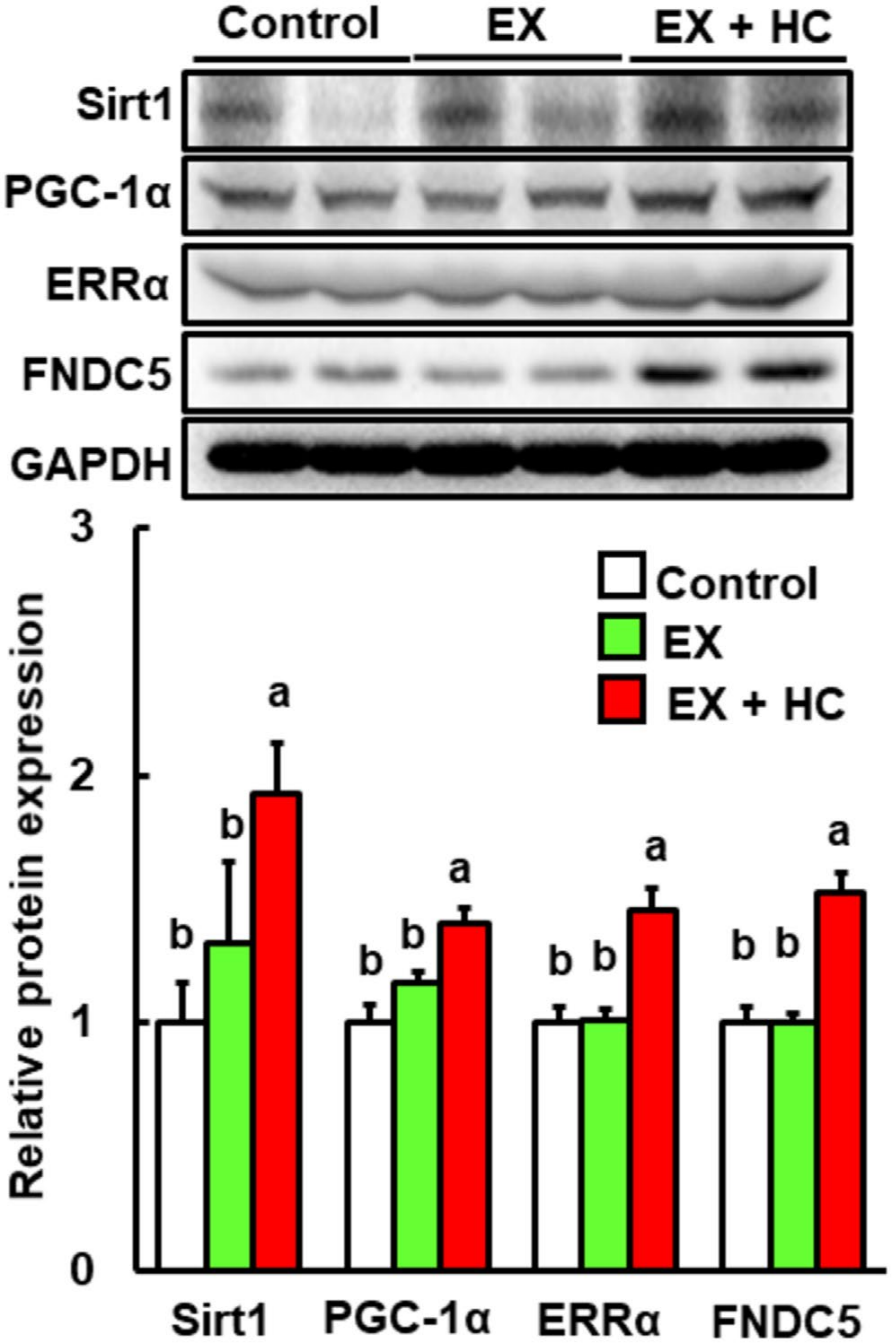
Protein expression levels of Sirt1, PGC‐1α, ERRα, FNDC5, and GAPDH in the hippocampus in the control, EX, and EX + HC groups. Protein levels are expressed as fold‐change relative to the control (= 1) after normalization to the GAPDH protein level. Data are presented as means + SEM (*n* = 6–7). Values without a common letter are significantly different at *p* < 0.05. EX, exercise; GAPDH, glyceraldehyde‐3‐phosphate dehydrogenase.

Previous studies indicated that exercise increases BDNF expression by inducing fibronectin type III domain‐containing 5 (FNDC5) via peroxisome proliferator‐activated receptor γ coactivator‐1α (PGC‐1α) and estrogen‐related receptor α (ERRα) (Wrann et al. 2013; Belviranlı and Okudan 2018). In addition, Cur ameliorated the depression‐like behavior accompanying the upregulation of the same molecules in rats. Furthermore, exercise‐induced increases in PGC‐1α expression are associated with upregulation of sirtuin 1 (Sirt1) (el Hayek et al. 2019). Therefore, this signaling pathway might help to induce BDNF by exercise and HC. As shown in Figure 5, the expression levels of these proteins (Sirt1, PGC‐1α, ERRα, and FNDC5) were significantly increased in the exercise + HC group but not in the exercise alone group compared with the control.

**FIGURE 6:**
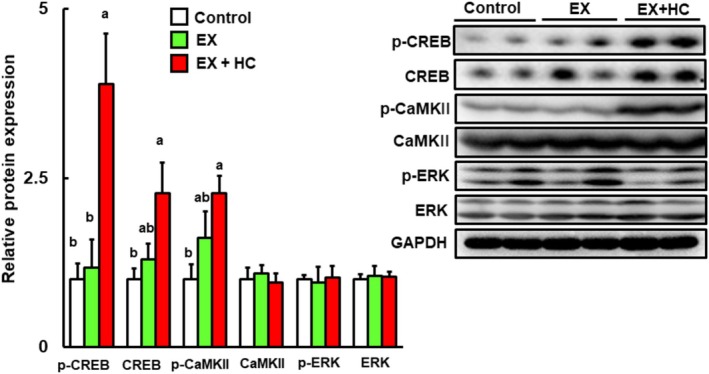
Protein expression levels of p‐CREB, CREB, p‐CaMKII, CaMKII, p‐ERK, ERK, and GAPDH in the hippocampus in the control, EX, and EX + HC groups. Protein levels are expressed as fold‐change relative to the control (= 1) after normalization to the GAPDH protein level. Data are presented as means + SEM (*n* = 6–7). Values without a common letter are significantly different at *p* < 0.05. EX, exercise; GAPDH, glyceraldehyde‐3‐phosphate dehydrogenase.

BDNF and PSD95 are among the target genes of cAMP‐response element‐binding protein (CREB); this activation is regulated by CREB phosphorylation (Suzuki et al. 2011; Chong et al. 2020). As shown in Figure 6, both phosphorylated CREB and total CREB levels were significantly higher in the exercise + HC group. Importantly, extracellular signal‐regulated kinase (ERK) and/or calcium/calmodulin‐dependent protein kinase II (CaMKII) participate in the phosphorylation of CREB (Chong et al. 2020; Yan et al. 2016; Zheng et al. 2023). Therefore, we examined whether CaMKII and/or ERK are phosphorylated in the exercise + HC group. The exercise + HC group exhibited significantly elevated expression of the phosphorylated CaMKII protein, whereas the expression level of phosphorylated ERK did not differ among the groups.

## 4 Discussion

Previous studies have reported that administration of Cur improves cognitive function (Cox et al. 2015, 2020; Kuszewski et al. 2020). However, some reports have shown that Cur failed to boost brain function, and the dose of Cur needs to be high because of its extremely low bioavailability (Santos‐Parker et al. 2018; Ringman et al. 2012). To overcome this problem, we used an amorphous individual dispersion technique to develop a novel highly bioavailable HC formulation for use as a dietary supplement (Sunagawa et al. 2021). We demonstrated strong absorption of HC in both rats and humans (with approximately 3.4‐fold higher absorption vs. another Cur formulation in humans) (Sunagawa et al. 2021). Here, we confirmed significantly higher bioavailability for HC than for NC in ICR mice of the same age. High doses of NC can lead to adverse effects such as liver damage (Liu et al. 2022). It has also been reported that a high dose of NC can decrease the effectiveness of drugs (Hussaarts et al. 2019). Although we did not perform comparative studies of adverse effects and drug interactions between HC and NC at the same dose, using HC at a lower dose compared with NC might avoid unexpected adverse effects or drug interactions.

As described in the introduction, exercise is known to improve cognitive function (Thomas et al. 2020; Yu et al. 2021). Therefore, we hypothesized that a combination of HC and exercise would effectively amplify the improvement in cognitive function and reduce the dose of Cur. After confirming that NC or HC alone did not improve cognitive function, we showed that exercise and HC in combination, but not NC, significantly improved spatial memory and learning, in contrast to exercise alone. Although HC alone (4.5 mg Cur/kg) did not significantly improve cognitive function, exercise combined with HC at the same dose significantly improved cognitive function without increasing the dose of HC. Therefore, exercise combined with HC has an advantage in that cognitive function can be significantly improved without increasing the dose of HC. We did not examine whether the same effect can be obtained with a 2‐ or 1‐week experiment as with a 4‐week experiment. We will need to verify whether similar results can be achieved with a shorter experimental period.

Next, we investigated how the combination of HC and exercise improved cognitive function. BDNF is one of the most important molecules for improving cognitive function in the hippocampus (Tyler et al. 2002). Here, the protein level of BDNF in the hippocampus was significantly increased in the exercise + HC group. Previous studies have reported that FNDC5 is involved in increased BDNF expression (Wrann et al. 2013; Belviranlı and Okudan 2018) and that the increase in BDNF expression is additionally linked to increases in the expression levels of Sirt1, PBC‐1α, and ERRα, which interact with PGC‐1α (el Hayek et al. 2019). In the present study, all of these proteins were significantly higher in the exercise + HC group versus control. Therefore, the Sirt1/PGC‐1α/FNDC5/BDNF pathway is involved in the cognitive function improvement with the exercise and HC combination. With regard to the pathway of BDNF upregulation, phosphorylation of CREB via CaMKII and/or ERK also affects BDNF expression (Chong et al. 2020; Yan et al. 2016; Zheng et al. 2023). In the present study, the expression levels of p‐CREB and p‐CaMKII were significantly elevated in the exercise + HC group. Therefore, the improvement in cognitive function with the combination of exercise and HC is associated with the combined activation of the Sirt1/PGC‐1α/FNDC5/BDNF pathway and CREB via CaMKII. In addition, knockout mice or siRNA‐mediated knockdown cells of members of the Sirt1/PGC‐1α/FNDC5/BDNF pathway may be a useful tool for further analyses and to confirm the mechanism. Although the present results were obtained based on protein levels and did not include knockout mice or knockdown cells, they provide significant insight into the mechanisms of BDNF‐related pathways influenced by exercise combined with HC.

Some limitations of this study should be noted. First, we confirmed that HC is more highly absorbable than NC in the plasma of ICR mice of the same age. However, because the hippocampus of mice is extremely small and it was difficult to measure exact Cur concentrations, we were unable to obtain data on Cur distribution in the hippocampus. If significantly more Cur accumulates in the hippocampus with HC than with NC, this may be an important finding for the utilization of HC. One previous article reported that Cur distributed to the brain stem, striatum, and hippocampus after the intravenous injection of a Cur formulation to rats (Chiu et al. 2011). These results suggest that the combination of exercise and HC may not only improve learning and memory, but also be effective for other brain disorders. In addition, it remains unclear whether Cur itself is involved in the improvement in cognitive function when combined with exercise or whether the metabolites of Cur play a greater role. Although previous studies reported that Cur metabolites underlie its physiological functions in vitro (Harada et al. 2022), we could not measure the metabolites of Cur in the plasma and hippocampus. Second, we did not examine whether acetylcholine participates in the cognitive function‐enhancing effects of exercise and HC. Because the decline in cognitive function with age and Alzheimer's disease is linked to lower levels of acetylcholine in the brain (Micheau and Marighetto 2011; Hung and Fu 2017), an increase in the acetylcholine concentration in the brain with exercise and HC may also contribute to the enhanced cognitive function. Third, it is necessary to examine in detail whether crosstalk between the skeletal muscle and brain participates in the improved cognitive function in the exercise + HC group. Exercise increases the expression of FNDC5 in skeletal muscle, and irisin, which is cleaved from FNDC5, crosses the blood–brain barrier to reach the brain and increase BDNF expression (Jodeiri Farshbaf and Alviña 2021; Maak et al. 2021). However, no significant difference was detected in the skeletal muscle levels of FNDC5 among the groups (Figure S2). Thus, the ability of exercise and HC to upregulate BDNF expression in the hippocampus is unlikely to be related to FNDC5/irisin from skeletal muscle. To obtain more evidence in this regard, skeletal muscle‐specific FNDC5 knockout mice could be a valuable tool for continued research.

**FIGURE 7:**
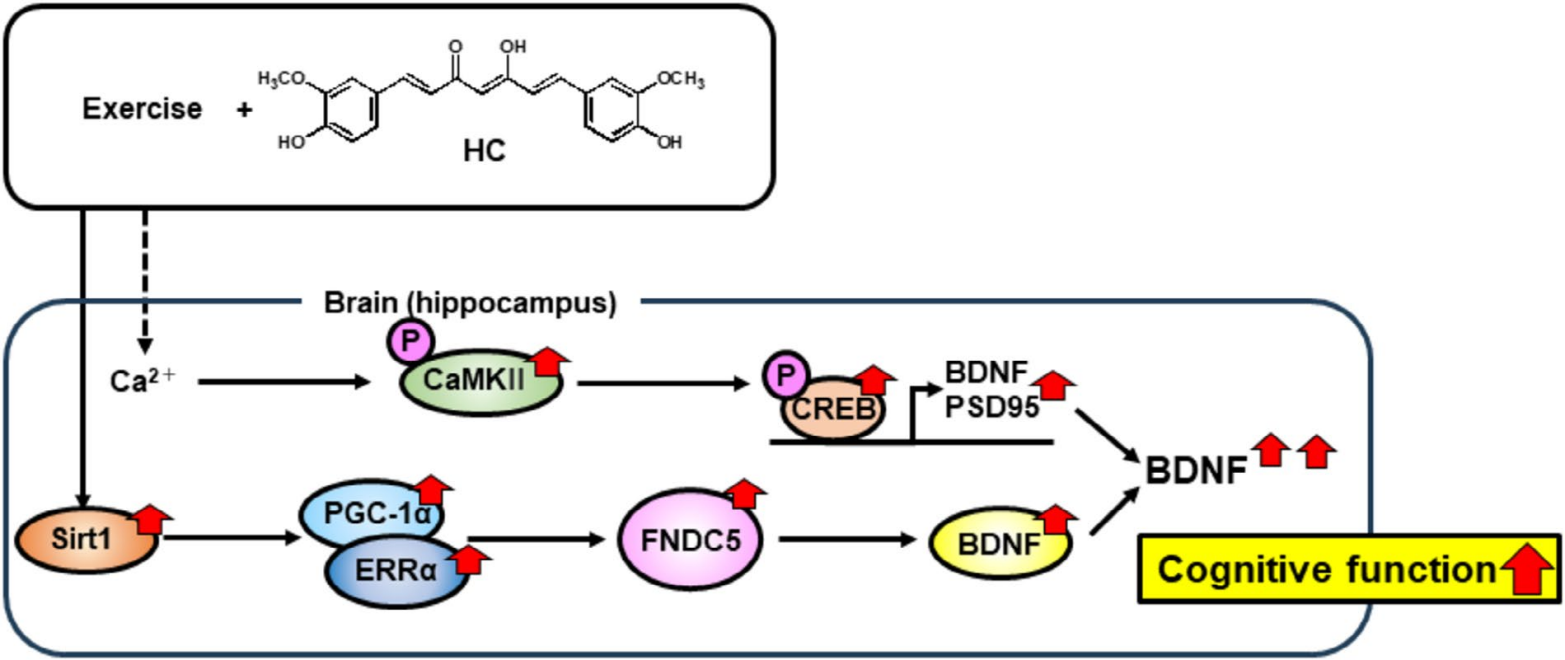
Proposed mechanism of cognitive improvement with combined exercise and HC. The Sirt1/PGC‐1α/FNDC5/BDNF pathway and activation of CREB via CaMKII enhance the upregulation of BDNF with exercise and HC.

In conclusion, HC, but not NC, combined with exercise improves learning and memory ability in mice, despite the lack of an effect of exercise alone. This effect is associated with upregulation of BDNF expression via activation of two related pathways (FNDC5 and CREB) (Figure 7). Our results suggest the possible ability of HC to boost learning and memory functions in both younger and older adults as a dietary supplement during exercise.

## Author Contributions

**Tomoya Suzuki:** conceptualization (supporting), investigation (lead). **Chisa Fushimi:** investigation (supporting). **Hiroki Aoyama:** investigation (equal), writing – original draft (supporting). **Atsuhiro Kishimoto:** investigation (supporting), writing – original draft (supporting). **Yasuhiro Katsuura:** investigation (supporting), writing – original draft (supporting), writing – review and editing (supporting). **Takanori Tsuda:** conceptualization (lead), data curation (lead), funding acquisition (lead), project administration (lead), supervision (lead), writing – original draft (lead), writing – review and editing (lead).

## Conflicts of Interest

H.A., A.K., and Y.K. are employees of Therabiopharma Inc.; the novel highly bioavailable Cur formulation and its placebo were supplied free of charge by Therabiopharma Inc.

## Supporting information


**Figure S1.** Schematic experimental design (A), time spent exploring the familiar and novel location (in seconds) (B) and recognition index (%) (C) after NLR test, and latencies (in seconds) after PA test (D) in the control, EX, and EX + NC groups. Data are presented as means ± SEM (*n* = 5–7). EX, exercise; NS, not significantly different.
**Figure S2**. The protein expression of FNDC5 in skeletal muscle in the control, EX, and EX + HC groups. Protein levels are expressed as fold‐change relative to the control (= 1) after normalization to the GAPDH protein level. Data are presented as means ± SEM (*n* = 6–7). EX, exercise; GAPDH, glyceraldehyde‐3‐phosphate dehydrogenase.

## Data Availability

The data that support the findings of this study are available from the corresponding author upon reasonable request.
