# Supplementary material for: Highly Bioavailable Curcumin, but Not Native Curcumin, Combined With Exercise Improves Cognitive Function in Mice
Source: Food Sci Nutr. 2025 Apr 30;13(5):e70219. doi: 10.1002/fsn3.70219 (PMC12041664; doi:10.1002/fsn3.70219)
Supplement: Supplementary file 1 — Figure S1. Schematic experimental design (A), time spent exploring the familiar and novel location (in seconds) (B) and recognition index (%) (C) after NLR test, and latencies (in seconds) after PA test (D) in the control, EX, and EX + NC groups. Data are presented as means ± SEM (n = 5–7). EX, exercise; NS, not significantly different. Figure S2. The protein expression of FNDC5 in skeletal muscle in the control, EX, and EX + HC groups. Protein levels are expressed as fold‐change relative to the control (= 1) after normalization to the GAPDH protein level. Data are presented as means ± SEM (n = 6–7). EX, exercise; GAPDH, glyceraldehyde‐3‐phosphate dehydrogenase. [file FSN3-13-e70219-s001.docx]

**Supporting information**

**Highly bioavailable curcumin, but not native curcumin, combined with exercise improves cognitive function in mice.**

**Authors: Tomoya Suzuki, Chisa Fushimi, Hiroki Aoyama, Atsuhiro Kishimoto,**

**Yasuhiro Katsuura and Takanori Tsuda**

**1. Supporting materials and methods**

The antibodies used in this study are shown in the tables below.

*Immunoblot analysis.* The tissue samples were homogenized, centrifuged and the total protein concentrations of the obtained supernatant were determined using a Protein Assay System (Bio-Rad, Richmond, CA) with bovine γ-globulin employed as a standard. Aliquots of the supernatant were treated with Laemmli sample buffer for 5 min at 100 °C. The samples were then loaded onto an SDS-PAGE system. The resulting gel was transblotted onto a PVDF membrane, which was blocked with 5% skim milk for 1 h at room temperature. After a washing with 20 mM Tris-HCl-buffered saline containing 0.05% (w/v) Tween 20 (TTBS), the membrane sheets were reacted with various antibodies for 16 h at 4 °C. After a washing with TTBS, the membranes were reacted with horseradish peroxidase-conjugated anti-rabbit IgG secondary antibodies (1:2000 dilution; Cell Signaling Technology, Tokyo, Japan) for 1 h at room temperature. After a washing, immunoreactivity was visualized using the ECL reagent (Thermo Fisher Scientific, Yokohama, Japan), and the relative signal intensity was evaluated with iBright CL1500 Imaging System (Thermo Fisher Scientific).

**3. Supporting Tables.**

**4. Supporting Figures.**


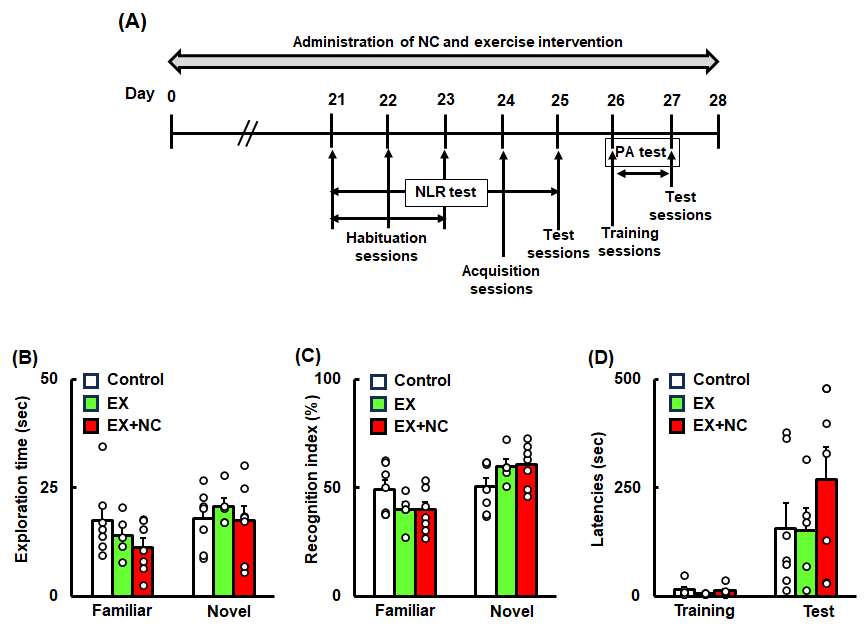


**Figure S1.** Schematic experimental design (A), time spent exploring the familiar and novel location (in seconds) (B) and recognition index (%) (C) after NLR test, and latencies (in seconds) after PA test (D) in the control, EX and EX + NC groups. Data are presented as means ± SEM (*n* = 5-7). EX, exercise; NS, not significantly different.


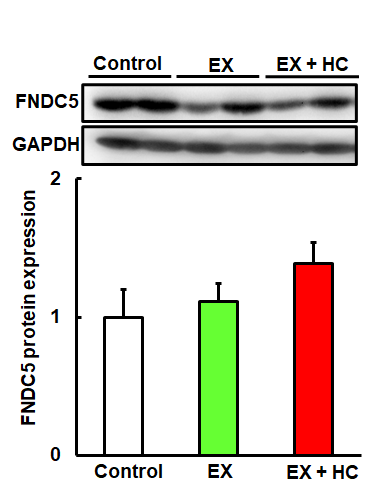


**Figure. S2.** The protein expression of FNDC5 in skeletal muscle in the control, EX and EX + HC groups. Protein levels are expressed as fold-change relative to the control (= 1) after normalization to the GAPDH protein level. Data are presented as means ± SEM (*n* = 6-7). EX, exercise; GAPDH, glyceraldehyde-3-phosphate dehydrogenase
